# Traditional values of virginity and sexual behaviour in rural Ethiopian youth: results from a cross-sectional study

**DOI:** 10.1186/1471-2458-8-9

**Published:** 2008-01-09

**Authors:** Mitike Molla, Yemane Berhane, Bernt Lindtjørn

**Affiliations:** 1 Centre for International Health, University of Bergen, Armauer Hansen Building, 5021, Bergen, Norway; 2 School of Public Health, Addis Ababa University, Addis Ababa, Ethiopia; 3 Addis Continental Institute of Public Health, Addis Ababa, Ethiopia

## Abstract

**Background:**

Delaying sexual initiation has been promoted as one of the methods of decreasing risks of HIV among young people. In traditional countries, such as Ethiopia, retaining virginity until marriage is the norm. However, no one has examined the impact of this traditional norm on sexual behaviour and risk of HIV in marriage. This study examined the effect of virginity norm on having sex before marriage and sexual behaviour after marriage among rural Ethiopian youth.

**Methods:**

We did a cross-sectional survey in 9 rural and 1 urban area using a probabilistic sample of 3,743 youth, 15–24 years of age. Univariate analysis was used to assess associations between virginity norm and gender stratified by area, and between sexual behaviour and marital status. We applied Kaplan-Meier and Cox regression analysis to estimate age at sexual debut and assessed the predictors of premarital sex among the never-married using SPSS.

**Results:**

We found that maintaining virginity is still a way of securing marriage for girls, especially in rural areas; the odds of belief and intention to marry a virgin among boys was 3–4 times higher among rural young males. As age increased, the likelihood of remaining a virgin decreased. There was no significant difference between married and unmarried young people in terms of number of partners and visiting commercial sex workers. Married men were twice more likely to have multiple sexual partners than their female counterparts. A Cox regression show that those who did not believe in traditional values of preserving virginity (adjusted hazard ratio [AHR] = 2.91 [1.92–4.40]), alcohol drinkers (AHR = 2.91 [1.97–4.29]), Khat chewers (AHR = 2.36 [1.45–3.85]), literates (AHR = 18.01 [4.34–74.42]), and the older age group (AHR = 1.85 [1.19–2.91]) were more likely to have premarital sex than their counterparts.

**Conclusion:**

Although virginity norms help delay age at sexual debut among rural Ethiopian youth, and thus reduces vulnerability to sexually transmitted infections and HIV infection, vulnerability among females may increase after marriage due to unprotected multiple risky sexual behaviours by spouses. The use of preventive services, such as VCT before marriage and condom use in marriage should be part of the HIV/AIDS prevention and control strategies.

## Background

HIV and AIDS have been a threat to Ethiopia since the mid-1980s [1] and different efforts have been put in place to alleviate the problem. The national HIV and AIDS prevention strategy in Ethiopia emphasizes the provision of regular and adequate information on HIV and AIDS and distribution of condoms among young people with the aim of lowering vulnerability and risk [2]. However, the prevalence among young adults is high. In 2005, the unadjusted prevalence of HIV among rural youth, 15–24 years of age, was 2.4% [3].

Behavioural change interventions should focus on causes that put a population at risk [4]. Early sexual initiation may predispose young people to HIV as their chances of having several partners before marriage increases [5]. Therefore, delaying the age of sexual debut and increased condom use is recommended in preventing HIV infection for this age group [6]. In Zambia, behavioural interventions have led to a decline in the prevalence of HIV [7].

In many societies, premarital sex is a taboo, especially among unmarried girls [8]. This norm is widespread in rural Ethiopia, as in many traditional societies [9]. A study about the concepts of HIV and AIDS in Ethiopia suggested that premarital sex may contribute to the expansion of HIV and AIDS [10]. Indeed, several studies from Ethiopia have shown that young people are engaged in premarital sex, have multiple sexual partners, and do not use condoms at all or use them irregularly [11-18].

Most of the studies conducted in Ethiopia represent school youth in urban settings, and often exclude people between 20 and 24 years of age and young married people [12-14,18,19]. Studies related to cultural causes are rarely mentioned [10,16,20,21]. There is a scarcity of rural-based studies in this area [22]. We did this study to assess the effect of traditional norms of virginity on premarital sex and risky sex in marriage in a rural setting of Ethiopia.

## Methods

### Design, sampling, settings, and participants

We did a cross-sectional study in nine rural and one urban area in the Butajira Rural Health Programme (BRHP) demographic and health surveillance site in south central Ethiopia in 2004 [23]. The study participants were youth, 15–24 years of age, living in the areas that were under surveillance. There were 10,475 young people, 15–24 years of age, living in the study site in 2004. Among these young people, 51.5% were females and 49.5% were males. Those 20–24 years of age comprise 44% of the population in which the majority (52.6%) were females.

We randomly selected 4,399 participants stratified, proportionate to the size of the 9 rural and 1 urban sites, using the BRHP database frame. The age and gender distribution of the sample was in accordance with the database, where 50.3% in which females and 44% of the youth were in the age group, 20–24 years. We used age reported by the respondents at data collection for the results.

Tracing of the individuals was possible using the database individual identifiers, such as individual identification numbers, household numbers, and name. The sampling method was aimed at obtaining a representative sample of sexually active youth [24]. Three thousand seven hundred forty-three young people participated in the study, giving a response rate of 85%. More girls 2044/2215 (92%) than boys 1699/2184 (78%) responded. The non-responses were mostly associated with absentees (427; 9.7%), out-migration from the study site (143; 3.3%), refusal (50; 1.1%), and wrong age, name, or gender because of database errors (36; 0.8%).

### Data collection procedures

We used a pre-tested and pre-coded questionnaire. The questionnaire was prepared in English and then translated into Amharic (the Ethiopian national language) and then back-translated into English by a language expert. We interviewed the study participants in Amharic. Interviewers who had completed high school and trained for five days collected the data. Same gender interviewers were used to decrease embarrassment as some of the questions were about personal sexual lifestyle issues. Interviews were conducted face-to-face in a household. We did a minimum of two revisits to absentees at the first visit. Two experienced supervisors and one field coordinator supervised the data collection. The collected questionnaires were checked every day and incomplete or inconsistent ones were sent back to the field for correction.

### Variables

The traditional norm of virginity was assessed using nine items. Beliefs about traditional values of virginity were determined based on the following question: "What is the attitude of the community you are living in towards preserving virginity until marriage? The response categories included three multiple choice responses: (1) virginity is encouraged/is the norm, (2) virginity is discouraged/not the norm, and (3) do not know. The responses were further dichotomized into two responses: (1) believe in the norm (for those who reported that the norm was encouraged) and (2) do not believe in the norm (for those who reported the norm was not encouraged and do not know). Beliefs about girls' and boys' virginity were determined by the following two questions: 1) Do you believe in the cultural value that girls should be virgins until marriage? and 2) Do you believe in the cultural value that boys should be virgins until marriage? The response categories were stated as "Yes" or "No." Intention of marrying a virgin for the never-married was determined in response to the following question: "Do you want to marry a virgin in the future?" The response categories were as follows: "Yes," "No," and "Indifferent." For those who reported to have been married, a question was posed if they had married a virgin as follows: "Did you marry a virgin girl/boy?" The response categories were as follows: "Yes," "No," and "Do not know."

Both boys and girls who reported to believe in the virginity norm were asked to give reasons for their belief. The response categories included five alternatives related to the prevention of HIV, sexually transmitted infections (STIs), and marital conditions, from which respondents could only choose one. The youth were asked to give their reasons and the enumerators marked according to the response. If the respondent does not respond to any choice on the list, the interviewers will read point-by-point. If the respondent does not agree with all the points mentioned, there was an option for other responses. For those who do not believe in preserving virginity, the reasons for their beliefs were stated in seven multiple choice statements where the same method of interviewing was followed as the former.

Sexual behaviour was assessed using the following items: sexual initiation among the never-married and age at sexual debut, reasons for sexual initiation, sex with casual partners, number of ever-partners, number of partners in the past 12 months, and whom the last sexual partner was for all participants. A question about sex with commercial sex workers was applied only for boys.

We assessed condom use by asking if the persons had ever used condoms in the past 12 months, during the most recent intercourse, or had used condoms when having sex with casual partners.

We used ever-alcohol use (home made or processesed) and khat chewing in the regression model due to the young age of our population.

### Analysis

We used Epi-info and SPSS for data analysis. Univariate analysis was used to assess the association between beliefs about the traditional norm of virginity with gender and residential area, and to assess the association of sexual behaviour with marital status.

We used Kaplan-Meier (KM) and Cox proportional hazard regression model to estimate age at sexual debut and to asses the predictors of premarital sex. Age of those who never had sex and age at sexual debut for the non-virgins was the time variable for the survival function. Sexual debut was taken as a failure variable while not having sex was censored. Proportional hazard ratios (HR) and 95% confidence intervals (CI) were calculated for risk factors of premarital sex (traditional norm of virginity, alcohol drinking, and khat chewing controlling for socio-demographic factors, such as age, gender, literacy, and school attendance). Variables with significant association (P value < 0.05) were included in the model. Interaction was tested and no important interaction term was found.

### Ethical considerations

We obtained ethical approval from the Regional Committee for Medical Research Ethics in Norway and the Ethiopian National Ethical Review Committee. Each participant volunteered to take part in the study and their parents also agreed that we interview people younger than 18 years of age. To maintain confidentiality, we did not record the names of the respondents on the questionnaire. We did the interviews in an area with maximum privacy for the study respondents.

## Results

### Socio-demographic features

The socio-demographic features of the population showed that 2,044 (54.6%) were girls, 2,730 (72.9%) were rural residents, 2,835 (75.7%) were between 15 and 19 years of age with a mean age of 17.5 years (SD = 2.7), Islam was the dominant religion followed by 2,740 (73.2%) of the study population, 3,045 (81.4%) were never married, 1,031 (27.5%) had never been to school, 1,753 (47%) had completed primary education, and 2,040 (54.5%) young people were currently attending school (Table 1).

**Table 1:** Socio-demographic profile of young people aged 15–24 in Butajira, Ethiopia, 2004 (n = 3743)

Variable	Male [n (%) = 1699 (45)]	Female [n (%) = 2044 (55)]	Total n (%)
Age			
15–19	1310 (77.1)	1525 (74.6)	2835 (75.7)
20–24	389 (22.9)	519 (25.4)	908 (24.3)
Residence			
Rural	1249 (73.5)	1481 (72.5)	2730 (72.9)
Urban	450 (26.5)	563 (27.5)	1013 (27.1)
Religion			
Muslim	1254 (73.8)	1486 (72.7)	2740 (73.2)
Christians	445 (26.2)	558 (27.3)	1003 (26.8)
Marital status			
Never married	1584 (93.2)	1461 (71.5)	3045 (81.4)
Ever married	115 (6.8)	583 (28.5)	698 (18.6)
Education			
Illiterate	260 (15.3)	771 (37.7)	1031 (27.5)
Primary	881 (51.9)	872 (42.7)	1753 (46.8)
Secondary	504 (29.7)	357 (17.5)	861(23.1)
Above highs school level	25 (1.5)	13 (0.6)	38 (1.0)
Read & write	29 (1.6)	31 (1.5)	60 (1.6)
Current attendance			
Attending	1087 (64.0)	957 (46.8)	2040 (54.5)
Out-of-school	616 (36.0)	1087 (53.2)	1703 (45.5)
Occupation			
Student	1072 (63.1)	919 (45.0)	1991 (53.2)
Unemployed	68 (4.0)	422 (20.6)	490 (13.1)
Farmer	434 (25.6)	7 (0.3)	441 (11.8)
Housewives	0	415 (20.3)	415 (11.1)
Employed (government and private)	125 (7.3)	281 (13.7)	406 (10.8)

### Beliefs about virginity

Urban and rural comparison showed that rural youth were three times more likely to believe in the traditional norm of remaining virgin until marriage than the urban youth (crude OR [95%CI] = 3.10 [2.46–3.89]). A gender-stratified comparison showed that both urban and rural young females were more likely to believe in the traditional norm of virginity than their male counterparts (crude OR [95%CI] = 6.3 [4.01–9.85] and 3.32 [2.29–4.48], respectively). Rural males were three times more likely to intend to marry a virgin than their urban counterparts (crude OR [95%CI] 3.89 [1.43–10.52]). Sex-stratified analysis showed that rural males more than rural females and urban females than urban males intend to marry a virgin (crude OR [95%CI] 0.51 [0.32–0.79] and 1.18 [1.17–2.78], respectively). The reason for keeping the virginity of boys and girls was related to marital condition for both rural and urban males than females in both areas (Table 2).

**Table 2:** Comparison of traditional values about virginity between residential area and gender among never-married youth in Butajira, Ethiopia, 2004 (n = 3045)

Variables	Rural male n (%)	Rural female n (%)	Rural female/rural male Crude OR and (95% CI	Urban male n (%)	Urban female n (%)	Urban female/urban male Crude OR and (95% CI)
Belief in traditional norm about virginity						
Virginity is not the norm	143 (12)	23(2)	1.00	126 (29)	46 (11)	1.00
Virginity is the norm	1008 (88)	1020 (98)	6.30 (4.01–9.85)	307 (71)	372 (89)	3.32 (2.29–4.48)
Beliefs about girls virginity						
Do not believe in virginity	19 (1)	5 (1)	1.00	12 (3)	8 (2)	1.00
Believe in virginity	1135 (99)	1038 (99)	3.48 (1.22–11.95)	421 (97)	410 (98)	1.46 (0.55–3.95)
Beliefs about boys virginity						
Do not believe in virginity	48 (4)	233 (22)	1.00	17 (4)	198 (35)	1.00
Believe in virginity	1103 (96)	810 (78)	0.15 (0.11–0.21)	416 (96)	270 (65)	0.06 (0.03–0.10)
Intention of marrying a virgin						
No/Indifferent	31 (3)	54 (5)	1.00	63 (15)	36 (9)	1.00
Yes	1120 (97)	989 (95)	0.51 (0.32–0.79)	370 (85	382 (91)	1.81 (1.17–2.78)
Perceived benefit of boys virginity (n = 2599)						
Pre condition for marriage	561 (51)	310 (38)	1.00	230 (55)	95(35)	1.00
Protects partners from HIV/STIs	542 (49)	500 (62)	1.67 (1.38–2.02)	186 (45)	175 (65)	2.28 (1.64–3.16)
Perceived benefit of girls virginity (n = 3004)						
Pre condition for marriage	590 (52)	490 (47)	1.00	233 (55)	194 (47)	1.00
Protects partners from HIV/STIs	545 (53)	548 (53)	1.21 (1.02–1.43)	188 (45)	216 (53)	1.38 (1.05–1.83)

Among the married, females were more likely to remain virgins until marriage than males in both rural and urban areas (crude OR [95%CI] 6.65 [2.70–16.27] and 13.8 [4.0–47.0], respectively). Of those who did not believe in boys' abstinence, 430 (67%) young people said it is difficult for a boy to abstain until marriage, while 34 (76.1%) of those who do not believe in girls' abstinence reported the same (data not shown).

### Sexual behaviour

Eight hundred two (21%) of the study participants were sexually active. Of these, 104 (13%) were never-married. Among the never-married 3,045, 104 (3.4%) had initiated sex. The median age for sexual initiation among the never-married was 17 years. More young males than young females were involved in premarital sex (Table 3). Area-stratified analysis showed that 96/851 (11.3%) of the urban and 8/2194 (0.4%) of the rural never-married had initiated sex.

**Table 3:** Prevalence of premarital sexual activity and sexual behaviour among never-married youth in Butajira Ethiopia, 2004

Variable	Male	Female	Total n (%)
Ever had sex (n = 3045)			
Never (n and %)	1496 (51)	1445 (49)	2941 (97)
Yes (n and %)	88 (85)	16 (15)	104 (3)
Reasons for sexual initiation (n = 104)			
Personal desire (n and %)	49 (47)	9 (8)	58 (55)
Partner influence (n and %)	31 (30)	4 (4)	35 (34)
Peer influence (n and %)	7(7)	0	7 (7)
Rape (n and %)	0	3 (3)	3 (3)
Influenced by drugs and gifts (n and %)	1(1)	0	1 (1)
Number of partners in the last one year (n = 104)			
One (n and %)	84 (81)	16 (15)	100 (96)
More than one (n and %)	4 (4)	0	4 (4)
Ever use of condoms (n = 104)			
Yes (n and %)	68 (65)	7 (15)	75 (72)
No (n and %)	20 (19)	9 (9)	29 (28)
Condom use in the most recent sex (n = 75)			
Yes (n and %)	58 (77)	5 (7)	63 (84)
No (n and %	10 (13	2 (3)	12 (16)

**Figure 1:**
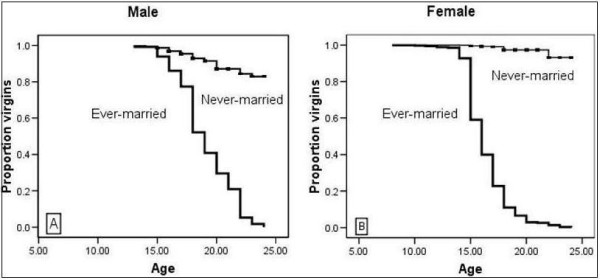
Kaplan-Meier indicating age at sexual debut among never-married and ever-married male and female youth in Butajira, Ethiopia.

Among the married 698, 96 (14%) males and 567 (81%) females reported that they had their first sexual experience when they get married at a median age of 16 years. Plots of the KM in Figure 1A indicate the proportion of young males who were married and those who were engaged in premarital sexual activity. The ever-married curve indicates that more young people initiate sex in marriage at a younger age. The never-married curve is higher than the ever-married curve with an increased downward slope after age 20 years, indicating that most young people did not engage in premarital sex at an early age. The wide gap between the ever-married and the never-married group suggests that premarital sex is not common in the study area. The age at sexual debut is lower among the married males (mean [95% CI] = 19 [18.5–19.4]) than the never-married males (mean [95% CI] = 23.2 [22.9–23.3]).

The KM plot for females in Figure 1B indicates that most young females become sexually active when they get married. The never-married curve is consistently higher with a very slight slope around age 22 years, indicating the proportion of girls having sex before marriage will increase late at an older age. On the other hand, the ever-married curve indicates that marriage for young females in the study area occurred at a young age. The mean age at sexual debut for the married young female was (mean [95% CI] = 16.3 [16.2–16.5]), while for those who had premarital sex, the mean age was higher (mean [95% CI] = 23.7 [23.5–23.9]). As age increased, the likelihood of remaining a virgin declined. The main reasons for sexual debut were a personal wish in 58 (56%) and partner influence in 35 (32%) of the never-married youth (Table 3).

Sexual behaviour was compared between the never-married and the married youth. There was no significant difference between marital status and having more than one partner in the past year before the study (crude OR [CI] 1.17 [0.39–3.46]). There was also no difference between the married and never-married young males in having sex with commercial sex workers (crude OR [CI] 0.80 [0.25–2.58]). The never-married were six times more likely to have sex with casual partners than were the married (crude OR [CI] 6.38 [2.40–16.93]). However, there was no difference in using a condom with casual partners among the married and the never-married youth. The never-married were more likely to use condoms ever and in the past year than their married counterparts (crude OR [CI] 0.02 [0.01–0.04] and crude OR [CI] 0.21 [0.08–0.53], respectively). However, there was no difference between the two groups in consistent use of condoms (crude OR [CI] 0.9 [0.30–2.65]; Table 4).

**Table 4:** Comparison of sexual behaviour between never-married and ever-married sexually active youth aged 15–24 in Butajira, Ethiopia, 2004 (n = 802)

Variable	Ever married [(n (%) = 698 (87)]	Never married [(n (%) = 104 (3)]	Crude OR and (95% CI) Never-married/Ever-married
Sex			
Female	583 (84)	16 (15)	1.00
Male	115 (16)	88 (85)	27.9 (14.77–49.73)
Sex with commercial sex workers [(n = 203) 115 married and 88 never-married male]			
No	107 (93)	83 (94)	1.00
Yes	8 (7)	5 (6)	0.80 (0.25–2.58)
Sex with casual partners			
No	689 (99)	96 (92)	1.00
Yes	9 (1)	8 (8)	6.38 (2.40–16.93)
Number of partners last 12 months			
One	675 (97)	100 (96)	1.00
>One	23 (3)	4 (4)	1.17 (0.39–3.46)
Ever use of condom			
Yes	47 (7)	75 (72)	1.00
No	651 (93)	29 (28)	0.02 (0.01–0.04)
Condom use in the past one year [(married: n = 47 and Never-married n = 75)]			
Consistent use	9 (19)	30 (40)	1.00
Inconsistent use	8 (17)	24 (32)	0.9 (0.30–2.65)
Did not use	30 (64)	21 (28)	0.21 (0.08–0.53)
Condom use with casual partners (n = 17)			
Yes	6	6	1.00
No	3	2	0.67 (0.04–8.48)

Gender-stratified analysis among the married showed that males were twice more likely to have more than one sexual partner (crude OR [CI] 2.83 [1.17–6.84]) and had sex with casual partners (crude OR [CI] 6.58 [1.74–24.9]). However, there was no significant difference in condom use between both sexes (Table 5).

**Table 5:** Comparison of sexual behaviour between male and female married youth aged 15–24 in Butajira, Ethiopia, 2004 (n = 698)

Variable	Female n (%)	Male n (%)	Crude OR and (95% CI) Male/Female
Age			
15–19	167 (28.6)	16 (13.9)	1.00
20–24	416 (71.4)	99 (86.1)	2.4 (1.42–4.339)
Number of. partners in the past one year			
One	568	107	1.00
More than one	15	8	2.83 (1.17–6.84)
Sex with casual partners			
No	579 (99.3)	110 (95.7)	1.00
Yes	4 (0.7)	5 (4.3)	6.58 (1.74–24.89)
Ever use of condoms			
Yes	38 (6.5)	9 (7.8)	1.00
No	545 (93.5)	106 (92.2)	0.82 (0.38–1.47)
Condom use in the past one year			
Consistent use	6 (15.8)	3 (33.3)	1.00
Inconsistent use	6 (15.8)	2 (22.2)	0.67 (0.04–8.48)
Did not use at all	26 (26.0)	4 (44.4)	0.46 (0.05–6.37)
Condom use with casual partners			
Yes	3	3	1.00
No	1	2	2.0 (0.11–156.75)

### Predictors of premarital sex

Cox regression analysis showed that youth who did not believe in the traditional value of preserving virginity until marriage were twice as likely to initiate sex before marriage (adjusted hazard ratio and [95% CI] = 2.91 [1.92–4.40]). In like manner, the older age group (adjusted hazard ratio and [95% CI] = 1.85 [1.19–2.91]), alcohol drinkers (adjusted hazard ratio and [95% CI] = 2.91 [1.97–4.29]), Khat chewers (adjusted hazard ratio and [95% CI] = 2.36 [1.45–3.85]), and those who have completed a primary education and above (adjusted hazard ratio and [95% CI] = 18.02 [4.34–74.42]) were more likely to have premarital sex than their counterparts (Table 6).

**Table 6:** Cox regression indicating factors associated with premarital sex among the never-married youth aged 15–24, adjusted for socio-demographic factors (sex, age, literacy and school attendance) in Butajira, Ethiopia, 2004 (n = 3045)

Variable	Sexual debut		Crude HR and (95% CI)	Adjusted HR and (95% CI)
	No	Yes		
Sex				
Female	1445	16	1.00	1.00
Male	1496	88	5.07(3.10–8.60)	1.45 (0.80–2.62)
Age				
15–19	2607	45	1.00	
20–24	334	59	8.85 (6.09–12.85)	1.85 (1.19–2.91)
Literacy				
Illiterate	611	2	1.00	1.00
Literate	2330	102	12.85 (3.18–51.90)	18.01 (4.34–74.42)
School attendance				
In school	1965	54	1.00	1.00
Out of school	976	50	1.82 (1.24–2.65)	1.27 (0.82–1.96)
Belief in virginity norm				
Yes	2638	69	1.00	1.00
No	303	35	4.06 (2.74–6.00)	2.91 (1.92–4.40)
Alcohol use				
No	2445	54	1.00	1.00
Yes	495	50	4.24 (2.92–6.16)	2. 91 (1.97–4.29)
Khat				
No	2183	30	1.00	1.00
Yes	758	74	6.56 (4.32–9.95)	2.36 (1.45–3.85)

## Discussion

We found that both married and never-married young males similarly engaged in risky sexual behaviours. Married females were less likely to engage in risky sexual behaviours than married males. Condom use in marriage was rare for both married females and males. Premarital sex was uncommon in the study area. Young males and females, who believed in traditional norms of preserving virginity until marriage, were less likely to engage in premarital sex. Never-married youth who were literate, used alcohol, chewed Khat, and older in age were more likely to begin premarital sex than their counterparts.

The large sample size with a representative selection of participants minimizes the chances of selection bias. The use of a community-based study, and inclusion of married youth, often considered as adults in other studies, gives a broader picture of youth in the rural set-up.

Using face-to-face interviews for sensitive issues can invite social desirability bias and therefore underestimate sexual activity. In a rural set-up, it is likely that boys could overestimate and girls would underestimate their sexual activity [25]. Marriage in rural areas occurs at a young age; this can also decrease the magnitude of sexual practice before marriage in such settings.

Encouraging cultural norms that can play a positive role in the prevention of HIV was set as an essential policy action for HIV prevention [4]. In Ethiopia, keeping virginity until marriage used to be an accepted norm in most societies. This cultural value has eroded with the introduction of education and advanced age in marriage in urban areas where premarital sex is common [12,17,25]. Our study suggests that this cultural value is still intact in rural societies of Ethiopia. Here, only 3% of the never-married study participants reported having had premarital sex. This is low compared to studies conducted among high school students in the same area [25].

Marriage in rural Ethiopia takes place at a younger age, especially for girls. In our study, more girls than boys were married and most of them were married as virgins, suggesting the norm is respected in the society. Shack, writing about the Gurage social organization, indicated that premarital sex is rare among girls in the area since it makes arrangements of marriage difficult [26]. Our study also demonstrated that the main reason of preserving a girl's virginity was related to securing marriage. Stratification of the analysis indicated that boys were more likely than girls to report this norm was related to marital conditions. Rural boys were also more interested in marrying a virgin than the rural girls. This indicates the emphasis given towards a girl's virginity and the relative freedom of boys to have sex before marriage. The strong belief of girls, both in rural and urban areas, about the virginity norm indicates that this norm is well accepted among girls.

The fact that the urban never-married youth and literates were more likely to have premarital sex than the rural youth suggests that the virginity norm is in transition in urban areas. Exposure to urban areas and socialization due to educational reasons could lead to the early practice of sex. The age at sexual debut for the never-married is higher than the married ones. This suggests that marriages in rural areas occur at a young age and abstinence until marriage might be difficult as the age of marriage increases for educational and other urban-related reasons, as is reported by others [25]. The 2005 Demographic and Health Survey (DHS) report of Ethiopia also showed that women with some secondary education had their sexual debut five years later than women with no education [27].

Alcohol and Khat use were strongly associated with premarital sexual initiation. The leaves of the khat bush, grown in East Africa and in the Arabian peninsula, is chewed habitually by many people for its stimulating effect [28]. The effect resembles that induced by weak amphetamine. Habitual users report that it produces increased levels of energy, alertness, and self-esteem, sensations of euphoria, enhanced imagination, and the capacity to generate ideas [29,30]. Butajira is a khat-growing area, and many local residents become accustomed to khat as young as 8 years of age [31]. Rural youth coming to the urban centre for high school education often chew khat for socialization and to stay alert for educational reasons [31]. Alcohol drinking usually follows khat chewing and might be associated with unprotected sex [32].

We observed a similar pattern of having sex with commercial sex workers among young married and never-married males. Similarly, there was no difference between married and never married youth in having multiple partners in the past year where married men were more likely to have had multiple partners than married females. This shows the increased risk of introducing HIV into marriages in rural areas where the status of females is low. The 2005 DHS report indicated that the prevalence of HIV in the age group, 15–19 years, was 0.7% for females and 0.1% for males. Among the 20–24 year olds, the prevalence among females was 1.7% and 0.4% for males. This age and sex pattern suggests that young females are more vulnerable than young males as they start sex earlier and usually with older males. The rural prevalence of the region (Southern Nation Nationalities and Peoples Region [SNNPR]), where this study was conducted was 0.2%. [27]. A verbal autopsy study conducted in the study area showed that 10.8 and 3.7% of the deaths in the urban and rural areas were attributable to HIV, respectively [33].

In our study area, 28% of the females were in a polygamous marital union and females believe that their husbands spread STIs because of the mobility of males to the urban areas [34]. The migratory nature of young adults could worsen the problem as the chances of seeing a prostitute is high in the urban areas. Young males, who migrate to the urban area for trading purposes, visit their families back home for holidays [35]. Studies from Bangladesh and South Africa have suggested that sexual behaviour of married males living away from home may put themselves and their spouses at risk of HIV [36,37].

A study on early marriage and HIV risks across sub-Saharan Africa showed that married girls were at a higher risk of HIV than their unmarried counterparts. Further, the partners of unmarried girls were at lower risk of HIV than the spouses of married girls [38].

Condom use with casual partners was practiced similarly among married and never-married youth compounded the risk of HIV in marriage. On the other hand, the low use of condoms among young married people is based on the deep-rooted belief of considering marriage as a protection against HIV [27,39]. Marriage places young girls at a higher risk of HIV as their ability of abstaining or using condoms is limited. The higher frequency of sex among newlyweds, coupled with unprotected sex might make them more vulnerable to HIV [38].

## Conclusion

Delaying sex is an important means of preventing HIV and other STIs among young people. Strengthening the norm of virginity is helpful in rural settings where access to information and condoms is limited. However, it makes young females more vulnerable as they start unsafe sex during marriage and yet consider marriage to be a guarantee of protection against STIs, including HIV. In addition, it increases the risks of young women by restricting them from getting information and health services out of fear of being considered to be sexually active. Therefore, this cultural norm should be propagated together with health education about condom use when delaying sex is not possible. Both married and unmarried youth (males and females) should be aware of the risk of HIV in marriage. Voluntary counselling and testing before marriage, faithfulness, and condom use in marriage should go hand-in-hand in health education programs. Further longitudinal studies examining the risk of HIV in marriage and potential use of female condoms in a rural set-up is recommended.

## Competing interests

The author(s) declare that they have no competing interests.

## Authors' contributions

All authors participated in the design of the study. MM and YB participated in the data collection and follow-up. MM and BL analysed the data. All authors participated in the drafting and approval of the manuscript.

## Pre-publication history

The pre-publication history for this paper can be accessed here:


